# Editorial: Desiccation tolerance in land plants: from mechanisms to evolution

**DOI:** 10.3389/fpls.2023.1210946

**Published:** 2023-06-27

**Authors:** Xiaoshuang Li, Bei Gao, Andrew J. Wood, Julia Buitink, Daoyuan Zhang, Melvin J. Oliver

**Affiliations:** ^1^ State Key Laboratory of Desert and Oasis Ecology, Key Laboratory of Ecological Safety and Sustainable Development in Arid Lands, Xinjiang Institute of Ecology and Geography, Chinese Academy of Sciences, Urumqi, China; ^2^ Xinjiang Key Laboratory of Conservation and Utilization of Plant Gene Resources, Xinjiang Institute of Ecology and Geography, Chinese Academy of Sciences, Urumqi, China; ^3^ Department of Plant Biology, Southern Illinois University-Carbondale, Carbondale, IL, United States; ^4^ Université d’Angers, Institut Agro, INRAE, IRHS, SFR QUASAV, Angers, France; ^5^ Division of Plant Sciences and Technology, Interdisciplinary Plant Group, University of Missouri, Columbia, MO, United States

**Keywords:** desiccation tolerance, editorial, special topic, resurrection plants, evolution

Desiccation tolerance (DT) is the ability to survive and recover from the equilibration of the water potential of cellular contents to that of the surrounding air. Most plants resist water loss and have evolved strategies to maintain their cellular water potential above that of the surrounding air and are desiccation sensitive with cellular water potentials of -3 to -6 MPa generally lethal. Desiccation-tolerant plants allow the equilibration of the water potential of their cells to that of the air and can tolerate tissue water potentials of -100 MPa and lower (Alpert and Oliver, 2002). Most seeds, pollen and spores are also desiccation tolerant.

Vegetative desiccation tolerance (VDT) was present in early bryophytes (Oliver et al., 2005), suggesting acquisition of desiccation tolerance was critical for the colonization of the land by primitive plants (Mishler and Churchill, 1985). Phylogenetic evidence indicates that VDT was lost during the evolution of tracheophytes but has reappeared in the lycophyte, fern, and angiosperm lineages (Mishler and Churchill, 1985; Oliver et al., 2020). Desiccation tolerance in seeds (SDT) appeared subsequent to its loss during tracheophyte evolution. SDT differs from VDT in that it is part of a developmental program that directs loss of water during seed maturation. SDT, along with dormancy, enabled both gymnosperms and angiosperms to disperse and establish in almost every terrestrial ecosystem. VDT in angiosperms evolved in at least thirteen lineages spanning both dicots and monocots (Gaff and Oliver, 2013) and there is growing evidence that VDT in the angiosperms evolved from SDT by a ‘rewiring’ of the controlling genetic networks (Farrant and Moore, 2011; VanBuren, 2017).

SDT was critical for the rise of civilization and global human expansion *via* its role in plant domestication (Diamond, 2002; Purugganan, 2019; Schaal, 2019). The continued importance of DT, both seed and vegetative, lies in understanding the mechanisms that enable cells to withstand dehydration and recover (Oliver et al., 2020). The hope is that within these processes novel strategies for improving crop stress tolerance will emerge: a vital need for a changing climate. The greatest promise comes from investigations into the genomic aspects of desiccation tolerance and recent whole genome sequencing of desiccation tolerant plants (http://desiccation.egi.ac.cn/)offer new insights into both the evolution of DT and the mechanisms and gene networks that control it (González-Morales et al., 2016; VanBuren et al., 2018).

Although we have made significant progress in understanding how plants tolerate desiccation (Oliver et al., 2020) there is still much to learn and many questions to be answered. It was with this in mind that we selected the topic of ‘*Desiccation tolerance in land plants: from mechanisms to evolution*’ for this Research Topic.

In an enlightening review of the studies of the seeds of the xerophytic plant *Caragana korshinski*, Peng et al. built a strong case for the use of xerophytic seeds for uncovering the underlying mechanisms of SDT. They also advocated for the positive aspects of re-establishment of DT in germinating seeds as a means for studying important regulatory aspects of DT. Staying with SDT, Sano et al. investigated chromatin dynamics during the short post-germination period in *Medicago trunculata* seeds when desiccation tolerance can be re-induced by a PEG treatment before being irretrievably lost as the seedling develops. They provided convincing evidence that PEG stimulated a more open configuration of the chromatin encompassing a number of induced DT related genes. They also demonstrated that an increase in H3K27me3 marking was associated with termination of the developmental window within which DT could be re-induced.

Barthlott et al. demonstrated biofilms of the desiccation-tolerant cyanobacterium *Hassallia byssoidea* are superhydrophobic (repels water), a property that enhances gas exchange and excludes competitors. Superhydorphobicity, restricted to biological surfaces, was suggested to be an evolutionary innovation during the colonization of the land. The presence of the superhydrophobic surface of a cyanobacterial biofilm suggested that this property may have an early prokaryotic Precambrian origin. They demonstrated the presence of superhydrophobic surfaces in all land plants and offered an argument for rethinking the role of superhydrobicity in plant evolution.

Three of the remaining articles focused on desiccation tolerance in bryophytes. Yang et al. investigated a comprehensive time course analysis of the dehydrating and rehydrating transcriptomes of gametophytes of the DT biocrust moss *Syntrichia caninervis*. Their analysis highlighted the accumulation of transcripts related to cellular protection during desiccation, in particular transcripts of oxidative metabolism, and the decline in transcripts associated with photosynthesis. Rehydration reversed this trend. The analysis highlighted the importance of several transcription factor families in the desiccation rehydration response. Fang et al. investigated the integrative response of the transcriptome and metabolome of the Antarctic moss *Pohlia nutans* to a PEG imposed water deficit stress. Almost half of the differentially changed metabolites were flavonoids and lipids and those transcripts that integrated well with the metabolite data were for flavonoid and long-chain fatty acid biosynthesis genes. The third article focuses on evolution of the abiotic stress responsive C2H2-type zinc finger proteins (C2H2-ZFPs) along with a comparison of the expression characteristics of these gene in *Physcomitrium patens* and Arabidopsis during dehydration and rehydration. Li et al., in a comprehensive study, demonstrated a general increase in C2H2-ZFP genes as plant complexity increased, with *Physcomitrium* exhibiting a larger number than expected likely related to the whole genome duplication event that occurred in its evolutionary history. Expression profiles for the C2H2-ZFPs of *Physcomitrium* and Arabidopsis during dehydration and rehydration exhibited different patterns of transcript accumulation reflecting both the phylogenetic relationships and types of conserved promoter domains; Z-type in non-seed plants and Q-type in seed plants. These differences highlighted the desiccation tolerance and sensitivity of these two plants.

Our remaining manuscript from Liu et al. explored the potential of a dehydration responsive transcription factor, DREB A-5, from the desiccation tolerant moss *Syntrichia caninervis* for crop improvement. They demonstrated that ectopic expression of a moss 35S-DREB A-5 construct improved germination rates and seedling salt tolerance. Overexpression Arabidopsis lines had enhanced levels of antioxidant enzymes and increased transcript abundance of stress related genes, including the salt overly sensitive (SOS) gene transcripts, SOS 1, 2 and 3. They provide convincing evidence that the DREB 5-A improved salt tolerance in part by stimulating jasmonic acid production.

## Author contributions

All authors listed have made a substantial, direct, and intellectual contribution to the work and approved it for publication.
